# The role of planfulness for well-being, stress, and goal disruption during COVID-19

**DOI:** 10.3389/fpsyg.2024.1224451

**Published:** 2024-02-08

**Authors:** Wesley C. Ameden, Elizabeth Tricomi, Samantha J. Heintzelman

**Affiliations:** Department of Psychology, Rutgers University-Newark, Newark, NJ, United States

**Keywords:** planfulness, COVID-19, goal pursuit, well-being, stress, individual differences

## Abstract

*Planfulness* refers to an individual’s tendency to be future oriented, mentally flexible, and cognitively strategic when engaging with goals, and has been shown to predict goal completion. We investigated the relationships among planfulness, goal disruption, stress, and psychological well-being during the COVID-19 pandemic, which served as a unique setback context. We measured these constructs using the planfulness scale, an *ad-hoc* survey item probing goal disruption in the pandemic, the perceived stress scale, and the Warwick-Edinburgh Mental Wellbeing Scale, respectively. Participants were university students (*N* = 174; mean age 23.03, SD: 4.37; 77% female). Higher planfulness predicted lower goal-disruption, lower stress, and higher well-being during the pandemic, extending its benefits beyond the goal domain. High levels of planfulness did not protect against goal disruption among those participants in which the self-reported personal impact of the pandemic was highest. Differences in goal disruption across levels of planfulness were constrained to lower reported pandemic impact. However, the differences in psychological well-being and stress by levels of planfulness were retained even when self-reported perceptions of personal pandemic impact were high. More planful students maintained lower stress and higher psychological well-being than their less planful peers across levels of adversity. These findings suggest that even in extremely difficult contexts in which planfulness does not protect against goal disruption, it still confers personal benefits in terms of psychological health.

## 1 Introduction

The COVID-19 pandemic shook the world, leading to millions of deaths and a general disruption of daily life. Initial studies showed that COVID-19, along with associated distancing and lockdown measures put in place to mitigate its effects, has had a negative impact on mental health. In particular, the pandemic has been linked to increases in feelings of anxiety, depression, changes in sleep patterns (e.g., Machado et al., 2020; Bueno-Notivol et al., 2021), and a disruption in daily routines that can give life a sense of coherence and meaning (Mohideen and Heintzelman, 2023). Additionally, the pervasiveness and duration of the pandemic have likely led to chronic feelings of threat and fear (Van Bavel et al., 2020). The distancing required to slow the spread of the virus has interfered with the positive impacts of social connectedness, a key component of coping with difficult circumstances and reducing stress (Brooks et al., 2020; Van Bavel et al., 2020). In addition to battling self-isolation, students had to find ways to achieve their academic goals despite a drastic change in school environment once work was moved online. While students faced setbacks with COVID-19, they were required to engage in coping strategies to continue to pursue their goals and buffer against negative impacts on their mental health. The pandemic provided an opportunity to examine potential constructs which are protective against these negative impacts (Pigaiani et al., 2020).

One individual difference which may protect against some of the negative impacts of the pandemic is *planfulness,* a stable suite of behavioral tendencies surrounding how one interacts and engages with their goals (Ludwig et al., 2018). The current study was conceived to assess the impacts of the pandemic on goal-related and mental health outcomes in students and to investigate potential protective factors. Specifically, the study investigates (1) the relationship between planfulness and self-reported disruption of one’s goals, (2) the relationship between planfulness and the important psychological outcomes of stress and psychological well-being, and (3) the role of planfulness as a potential moderator in the relationship between COVID experiences and students’ psychological well-being, stress, and goal disruption during the pandemic.

### 1.1 Planfulness and goals

Planfulness sits within the conscientiousness domain of the Big-Five personality traits and is posited to be a relatively stable individual difference (Ludwig et al., 2018). Planfulness is composed of three sub-components: (1) Temporal orientation, which refers to the tendency to be attuned to the future consequences of present actions, (2) cognitive strategies, or situating behaviors in line with goals, and (3) mental flexibility, or the ability to anticipate and handle obstacles interfering with goal progress.

Planfulness scores are related to measures of grit and conscientiousness, but explain unique variance in self-reported ability to achieve one’s goals (Ludwig et al., 2018). Specifically, those higher in planfulness who set a personal goal and report on their progress have been shown to make more progress on their goals than those with lower levels of planfulness (Ludwig et al., 2018). Planfulness can predict objective goal-directed behavior as well. Among students who reported a physical activity goal, those higher in planfulness swiped their student ID card to access the campus gym more across a 20-week period compared to those lower in planfulness, even when controlling for conscientiousness and other related constructs (Ludwig et al., 2019). Planfulness was thus associated with taking goal-oriented actions across time.

While an individual’s level of planfulness relates to their pursuit of goals that are important to them, it is not yet known how levels of planfulness might relate to goals during a major setback such as that experienced during the pandemic. The COVID-19 pandemic represents a roadblock to goal pursuit. A vast majority of individuals reported that their goals became more difficult in the wake of the pandemic (Vowels et al., 2022), and they stopped believing that they could carry out their goals, often resulting in goal abandonment (Ritchie et al., 2021). A strong relationship between planfulness and grit could suggest that planfulness may also be able to predict an individual’s ability to persist in working toward goals when faced with a setback. Planning in general has been investigated as a coping strategy (e.g., “I’ve been thinking hard about what steps to take”) during setbacks, but its effects on mental health have been mixed (see Eisenbeck et al., 2022). Given that planfulness involves skills such as orienting to the future, deploying cognitive strategies for goal achievement, and displaying mental flexibility, it could serve as a bulwark in the face of severe disruption.

### 1.2 COVID-19, well-being, and stress

Subjective well-being is a construct encompassing satisfaction with one’s life, high positive affect, and low negative affect (Diener et al., 2017). High levels of subjective well-being are associated not only with mental wellness but also with better physical health and longevity (for a review, see Diener et al., 2017; Heintzelman and Tay, 2018). Stress, on the other hand, is negatively associated with well-being (e.g., Ng and Diener, 2022). Meaning in life, which relates and contributes to well-being, has been emphasized as an important coping mechanism and protective factor when faced with both extreme and everyday stressors (Ward et al., 2023).

The COVID-19 pandemic was a major worldwide stressor which wreaked havoc on mental health. Studies reported a surge in rates of depression (Bueno-Notivol et al., 2021; Giuntella et al., 2021), and many adolescents reported feelings of anxiety and changes in their well-being (Pigaiani et al., 2020). Along with general declines in overall well-being and reported happiness, there were fears about financial stability (VanderWeele et al., 2021) and academic outcomes for adolescents (Pigaiani et al., 2020). Additionally, stress specifically related to the COVID-19 pandemic negatively impacted meaning in life, consequently resulting in decreased well-being (Arslan and Allen, 2022).

As the long-term effects of the pandemic continue to be investigated, it will be easier to uncover the ways in which it is similar to, and different from, other stressors that we encounter. Interestingly, Zhang et al. (2021) analyzed social media posts and found that pandemic severity, based on the location of the user, negatively impacted well-being by increasing negative affect. The authors argued that the pandemic differed from other stressors due to the unique circumstances of the lockdown, including decreased time around friends and family outside the house and the inability to separate work and home life due to work-from-home measures (Zhang et al., 2021). While this pandemic may be different from more common, everyday stressors, previous work has shown that constructs such as meaning and well-being are related to extreme stressors (and specifically stress related to COVID-19) as well as common, everyday ones (Ward et al., 2023).

Another factor that can promote well-being is routine. Routines contribute to our sense of coherence and meaning in life (Heintzelman and King, 2019) and can serve as a buffer or protective factor in difficult times. Many people saw their typical routines disrupted during the pandemic, and the enactment of routines was even more predictive of meaning in life during pandemic lockdowns than in pre-pandemic contexts (Mohideen and Heintzelman, 2023). Many people also experienced a lack of physical activity relative to pre-pandemic times that was detrimental to mental health (Giuntella et al., 2021).

The COVID-19 pandemic thus represents an extreme stressor which led to decreases in well-being and a general decline in mental health. Research will continue to uncover these effects and shed light on factors which can help protect well-being in the face of a stressor.

### 1.3 Planfulness and well-being and stress

Previous work with planfulness has been limited to examinations of its relationships with outcomes entirely in the goal domain. However, planfulness may share relationships with other important psychological outcomes including stress and psychological well-being. Indeed, committing to and perceiving progress toward attainable personal goals over time has been shown to promote psychological well-being (Brunstein, 1993). Further, more concrete progress toward goals across a semester has also been tied to increases in well-being across this same time period (Sheldon and Kasser, 1998). This link between goal progress and well-being suggests that individual differences that promote goal progress or protect against goal disruption may also relate to well-being.

Furthermore, variables related to planfulness, including conscientiousness, relate to greater positive affect and life satisfaction, and can serve as a buffer against stress by promoting effective coping (Bartley and Roesch, 2011; Duckworth et al., 2012; Sun et al., 2018). Grit, a perseverance for long-term goals (Duckworth et al., 2007), is another construct tied to both planfulness and goal achievement, and has also been positively associated with psychological well-being (Arya and Lal, 2018) and negatively associated with stress (Lee, 2017). The current study adds to this literature by probing a potential relationship between planfulness and the psychological outcomes stress and psychological well-being. We hypothesized that planfulness would positively relate to psychological well-being and negatively relate to stress.

### 1.4 Current study

In the current study, we aimed to examine the relationship between planfulness, stress, psychological well-being, and perceived goal disruption during the COVID-19 pandemic. In addition to sharing relationships with goal pursuit, stress, and psychological well-being, planfulness may be an individual difference which could serve as a buffer for these outcomes in difficult setback situations. The current study is the first to look at planfulness as a potential protective resource. Specifically, we measured students’ psychological well-being, perceived stress levels, and goal disruption in the midst of the COVID-19 pandemic. We investigated the relationships between participants’ perceptions of the personal impact of the pandemic (a stressful situation or setback) and these outcome measures, and the influences of planfulness on these relationships.

In summary, the current study builds on previous planfulness research to make three central contributions. First, we move beyond the existing scope of examined correlates by testing relationships between planfulness and stress and psychological well-being to build an understanding of the psychological relevance of individual differences in planfulness. Second, we examine the relationship between planfulness and goal markers in the context of an extreme setback, namely, the COVID-19 pandemic. Finally, we test the potential protective power of planfulness in buffering against the negative psychological impacts of major life setbacks by examining planfulness as a moderator of the relationship between perceived pandemic impact and goal disruption, stress, and psychological well-being.

### 1.5 Hypotheses

We hypothesized first that higher planfulness scores would be correlated with higher levels of psychological well-being, lower stress, and lower reported goal disruption. We further hypothesized that students higher in planfulness would be more protected against the negative psychological impacts of COVID-19, and thus that planfulness would serve as a buffer against the effects of the pandemic on these constructs. This study thus provides more insight into how planfulness may be a protective factor during a real-life setback, as well as how it may be related to psychological outcomes in addition to goal-related outcomes.

## 2 Methods

### 2.1 Participants

We recruited a sample of 199 undergraduate Psychology students at Rutgers University-Newark to complete online surveys in exchange for extra credit in a course. Participants completed surveys at three timepoints throughout the semester. The current analyses focused on timepoint 2 (mid-semester) because this is when the primary constructs of interest were measured. Twenty-five participants did not complete the survey at timepoint 2, leaving *N* = 174 for the present study (*N* = 105 in the Fall and *N* = 69 in the Spring), age *M*(*SD*) = 23.03 (4.37). One hundred twenty-five participants (72%) identified as female, 35 (20%) as male, and 14 (8%) did not provide an answer. The option to write in an alternative gender identity was provided, but no participants selected this option. The sample was racially diverse: 52 (30%) participants identified as Hispanic/Latinx, 65 (37%) identified as White, 33 (19%) as Black, 24 (14%) as Asian, 1 (0.6%) as Native Hawaiian/Other Pacific Islander, 18 (10%) as “Other,” 19 (11%) selected that they preferred not to answer, and 14 (8%) did not answer. Newark was a COVID-19 hotspot throughout the pandemic (Armstrong and Tully, 2020; Okoh et al., 2020) and thus this sample represents a population that experienced the effects of the pandemic quite intensely. Furthermore, all students in the sample were attending their college classes in a fully remote setting. A post-hoc sensitivity analysis using G*Power 3.1 (Faul et al., 2009) indicated that given *N* = 174 and a desired power of 0.8, the current study was powered to detect effect sizes at least as large as *R*^2^ = 0.053.

### 2.2 Data collection and survey items

Data were collected virtually using Qualtrics (Qualtrics, Provo, UT). The surveys contained several existing and *ad-hoc* scales designed to measure constructs including the target variables of the perceived effects of the COVID-19 pandemic on one’s life, planfulness, goal-disruption, well-being, and stress. The current study was part of a larger project investigating the effects of the pandemic on goal-related and mental health outcomes in undergraduate students. This larger project took place over two semesters (Fall, 2020 and Spring, 2021) with separate cohorts of students. None of the variables of interest differed significantly between the two samples as measured using independent-samples t-tests: Pandemic Impact: *t*(143.64) = 0.75 *p* = 0.46; Planfulness: *t*(155.15) = 0.999, *p* = 0.32; Stress: *t*(160.47) = −0.55, *p* = 0.58; Psychological Well-being: *t*(161.61) = 1.18, *p* = 0.24; Goal Disruption: *t*(146.8) = −0.53, *p* = 0.60. Thus, the two student samples were combined into a single sample for all analyses. As a result, this sample contains students from mid-semester in Fall, 2020 and Spring, 2021.

#### 2.2.1 Perceived pandemic impact

The perceived impact of the COVID-19 pandemic on students’ lives was assessed using a question developed for the present study: “How much has the pandemic affected your life overall?” which was rated from 1 (*Affected very positively*) to 5 (*Affected very negatively*), thus making higher numbers indicative of a more negative impact, *M*(*SD*) = 3.56 (0.92). Assessing the effect of the pandemic on a person’s life is difficult, as it may have impacted life in different ways for different people (e.g., some people may have been affected by COVID-19 itself, some may have been affected by needing to work from home or losing their job, etc.). This question was designed to be a broad, subjective measure of how participants feel they have been affected overall by the pandemic. We opted for this broad, participant-defined approach rather than selecting particular impacts in a top-down manner, as this likely would have missed some of the subjective impact felt by the participants. Examples of objective effects might be contracting the virus or knowing family members who tested positive. The Perceived Pandemic Impact variable did not differ in those who reported having tested positive for COVID-19 (*t* (47.19) = −1.28, *p* = 0.21), and was not related to the number of people that a person knew who had tested positive (*r* = −0.03*, p* = 0.65). This measure was thus a face valid way of measuring the perceived, subjective impact of the pandemic, accounting for the fact that each person has likely been affected in different ways.

#### 2.2.2 Planfulness

We assessed planfulness using the previously validated Planfulness Scale (Ludwig et al., 2018). This 30-item measure refers to an individual’s proclivity for adopting specific goal-related cognitions and strategies when pursuing goals. It consists of three 10-item subscales: temporal orientation, (e.g., “I regularly spend time and energy now to get what I want in the future”), mental flexibility, (e.g., “I think about my goal when I encounter obstacles to achieving it”), and cognitive strategies, (e.g., “I tend to take big projects and break them down into small pieces”). Responses were rated on a 5-point scale from 1 (*Strongly disagree*) to 5 (*Strongly agree*), such that larger numbers indicate higher levels of planfulness. While the Planfulness Scale is comprised of these three subscales, it is intended to be used as a composite variable (Ludwig et al., 2018). Thus, a participant’s planfulness score is an average of the 30 items, *M*(*SD*) = 3.52 (0.52), α = 0.90.

#### 2.2.3 Goal disruption

To measure whether the pandemic had affected students’ progress on their goals, we created an *ad-hoc* scale of goal-disruption. Questions asked about the disruption of goals in different areas of life. These areas included academic (“How has the pandemic affected your immediate progress toward academic goals?”), health and fitness (“How has the pandemic affected your immediate progress toward health and fitness-related goals?”), and personal (“How has the pandemic affected your immediate progress personal goals (such as hobbies)?”). Separate questions asked about disruption of longer-term goals in those same areas (e.g., “How has the pandemic affected your ability to plan and pursue your long-term academic goals?”). Responses were rated on a 5-item scale from 1 (*Affected very positively*) to 5 (*Affected very negatively*), such that higher numbers indicate more disruption of goal progress. In order to capture the disruption of all kinds of goals, across domains and timescales, these subscales were collapsed into a single measure of goal disruption, *M*(*SD*) = 3.27 (0.75), α = 0.84.

#### 2.2.4 Well-being

Psychological well-being was assessed using the validated 14-item Warwick-Edinburgh Mental Wellbeing Scale (Tennant et al., 2007). This 10-item scale measures a variety of feelings of psychological well-being (e.g., “I’ve been feeling good about myself”). Responses were rated on a 5-point scale from 1 (*None of the time*) to 5 (*All of the time*), such that higher numbers correspond to higher levels of psychological well-being, *M*(*SD*) = 3.12 (0.78), α = 0.94.

#### 2.2.5 Stress

Stress was measured using the validated Perceived Stress Scale (Cohen et al., 1983). This 10-item scale measures the frequency of feelings of stress in the past month (e.g., “How often have you found that you could not cope with all the things you had to do?”). Responses were rated on a 5-point scale from 1 (*Never*) to 5 (*Very often*), such that higher numbers indicate higher perceived stress, *M*(*SD*) = 3.11 (0.73), α = 0.90.

### 2.3 Analyses

We used Pearson’s correlations to test our hypothesis that planfulness is negatively correlated with stress and goal disruption, and positively correlated with well-being. Additionally, we hypothesized that planfulness would play a moderating role in expected relationships between COVID-19 impact and goal disruption, stress, and well-being, such that students with high levels of planfulness might be buffered against these negative psychological consequences of the pandemic. We tested this prediction with moderation analyses using RStudio Team (2020) “lm” function (R Core Team, 2022) to assess whether planfulness modified relationships between pandemic impact and goal disruption, well-being, and stress. We also ran additional models including age and gender as covariates. To investigate the possibility that planfulness is related to well-being and stress *through* its relationship with goal disruption, an exploratory mediation analysis was conducted using Hayes’ PROCESS macro for R (Hayes, 2022) which tests for the presence of mediation using bootstrapped 95% confidence intervals. These analyses can be found in the Supplementary material for this manuscript (Supplementary Figures 1, 2).

## 3 Results

### 3.1 Correlations

We first examined correlations among our variables of interest (Table 1). Planfulness was positively correlated with psychological well-being and negatively correlated with stress, extending the web of beneficial outcomes linked to planfulness. Further, planfulness shared a modest negative correlation with goal disruption in the context of the COVID-19 pandemic, which extends the relevance of these cognitive tendencies to difficult life circumstances.

**Table 1:** Correlations for variables of interest.

	Perceived pandemic impact	Planfulness	Stress	Well-being	Goal disruption	Age
Planfulness	−0.01					
Stress	0.27***	−0.37**				
Well-being	−0.25***	0.40***	−0.80***			
Goal Disruption	0.44***	−0.18*	0.27***	−0.31***		
Age	0.11	0.14	−0.14	0.11	0.07	
Gender	−0.06	−0.06	−0.14	−0.12	−0.10	−0.15

Gender is coded as Male = 0, Female = 1. *p < 0.05; **p < 0.01; ***p < 0.001.

### 3.2 Planfulness as a moderator

Next, we performed a series of regression analyses to investigate the moderating role of planfulness in the relationships between perceived pandemic impact and goal disruption, stress, and psychological well-being. Pandemic impact and planfulness were mean-centered and their product was computed to form the interaction term. Additional models were used including age and gender as covariates. These variables did not emerge as significant predictors and the pattern of results was the same for all regression analyses, so here we report results from the models without age and gender included. This also allowed us to include participants who did not report a gender. For visualization, all interaction effects were plotted at ± 1 standard deviation for planfulness (e.g., Aiken and West, 1991; Dawson, 2014) to represent relationships between pandemic impact and the psychological outcome variables at high and low levels of planfulness.

**Figure 1:**
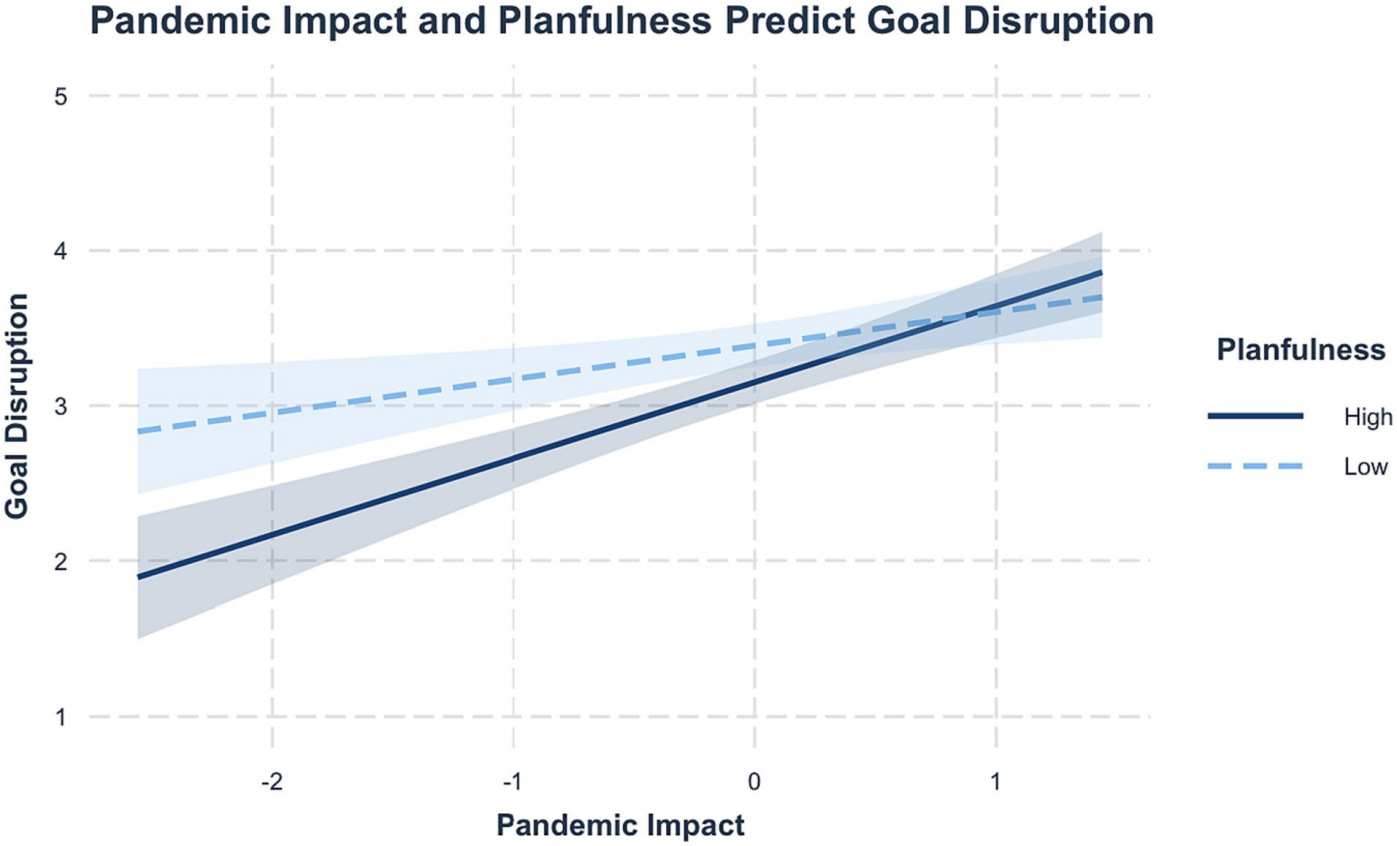
Pandemic impact and planfulness predicting goal disruption. Pandemic impact and planfulness are mean-centered, and shaded regions represent 95% CI. Highly planful students saw less goal disruption if pandemic impact was low, but this difference did not emerge when pandemic impact was high.

For goal disruption, there were main effects of pandemic impact (*β* = 0.35, *SE* = 0.54, *p* < 0.001) and planfulness (*β* = −0.22, *SE* = 0.10, *p* = 0.02), with high pandemic impact predicting higher goal disruption and high planfulness predicting lower goal disruption. Additionally, there was a significant interaction between pandemic impact and planfulness (*β* = 0.26, *SE* = 0.10, *p* < 0.01, *R*^2^ = 0.25). This interaction indicates that at low levels of pandemic impact, highly planful people experienced less disruption of their goals compared to less planful peers, but that at high levels of pandemic impact, there was higher goal disruption regardless of planfulness levels (Figure 1).

**Figure 2:**
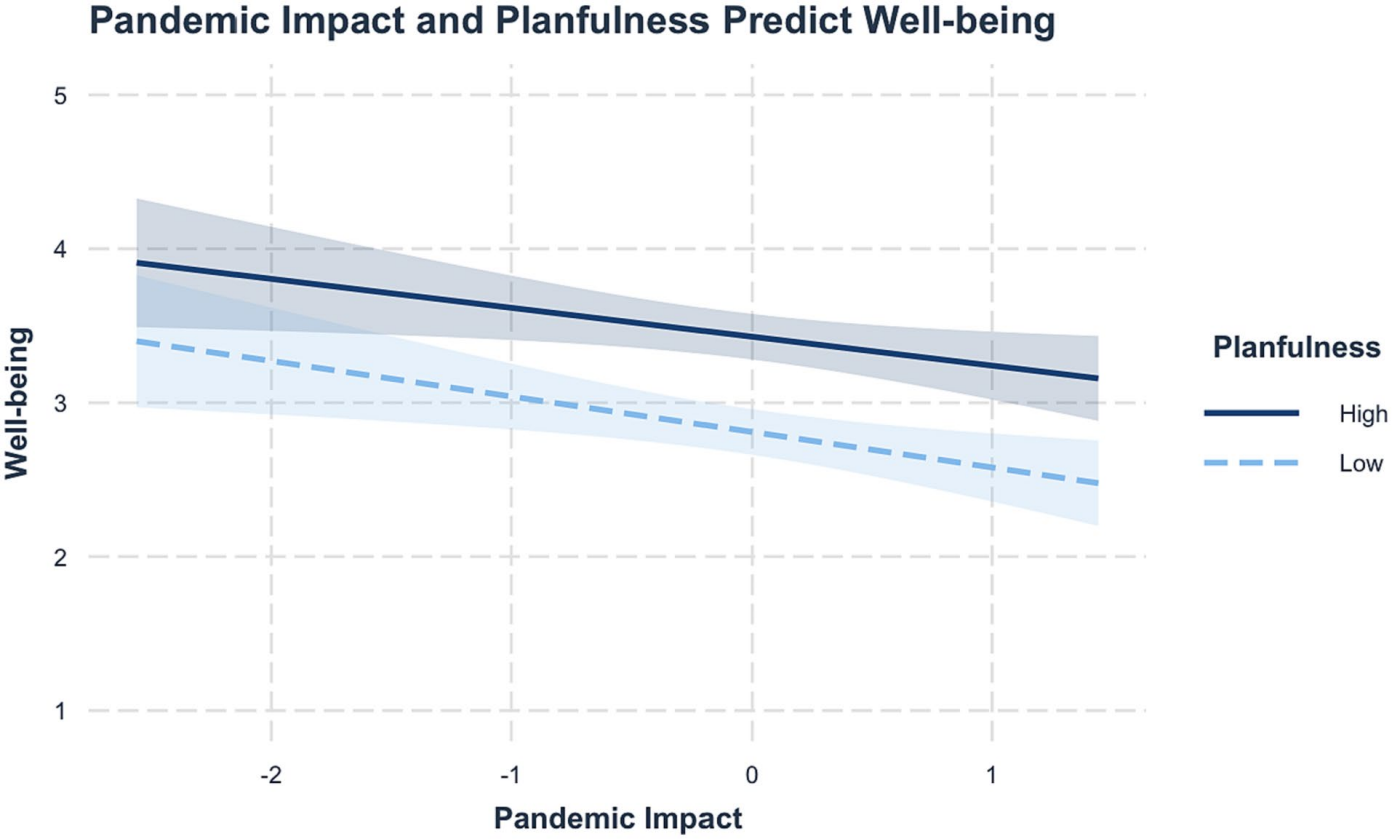
Pandemic impact and planfulness predicting well-being. Pandemic impact and planfulness are mean-centered, and shaded regions represent 95% CI. High pandemic impact was associated with lower well-being overall, and highly planful students maintained higher well-being than students low in planfulness regardless of pandemic impact levels.

For psychological well-being, there were main effects of pandemic impact (*β* = −0.21, *SE* = 0.06, *p* < 0.001) and planfulness (*β* = 0.59, *SE* = 0.10 *p* < 0.001), such that higher pandemic impact predicted lower psychological well-being, and higher planfulness predicted higher psychological well-being. There was no significant interaction between pandemic impact and planfulness (*β* = 0.04, *BSE* = 0.11, *p* = 0.70, *R*^2^ = 0.22). Higher levels of pandemic impact were associated with lower levels of psychological well-being regardless of planfulness levels, and individuals high in planfulness maintained higher psychological well-being compared to those with low planfulness whether pandemic impact was high or low (Figure 2).

**Figure 3:**
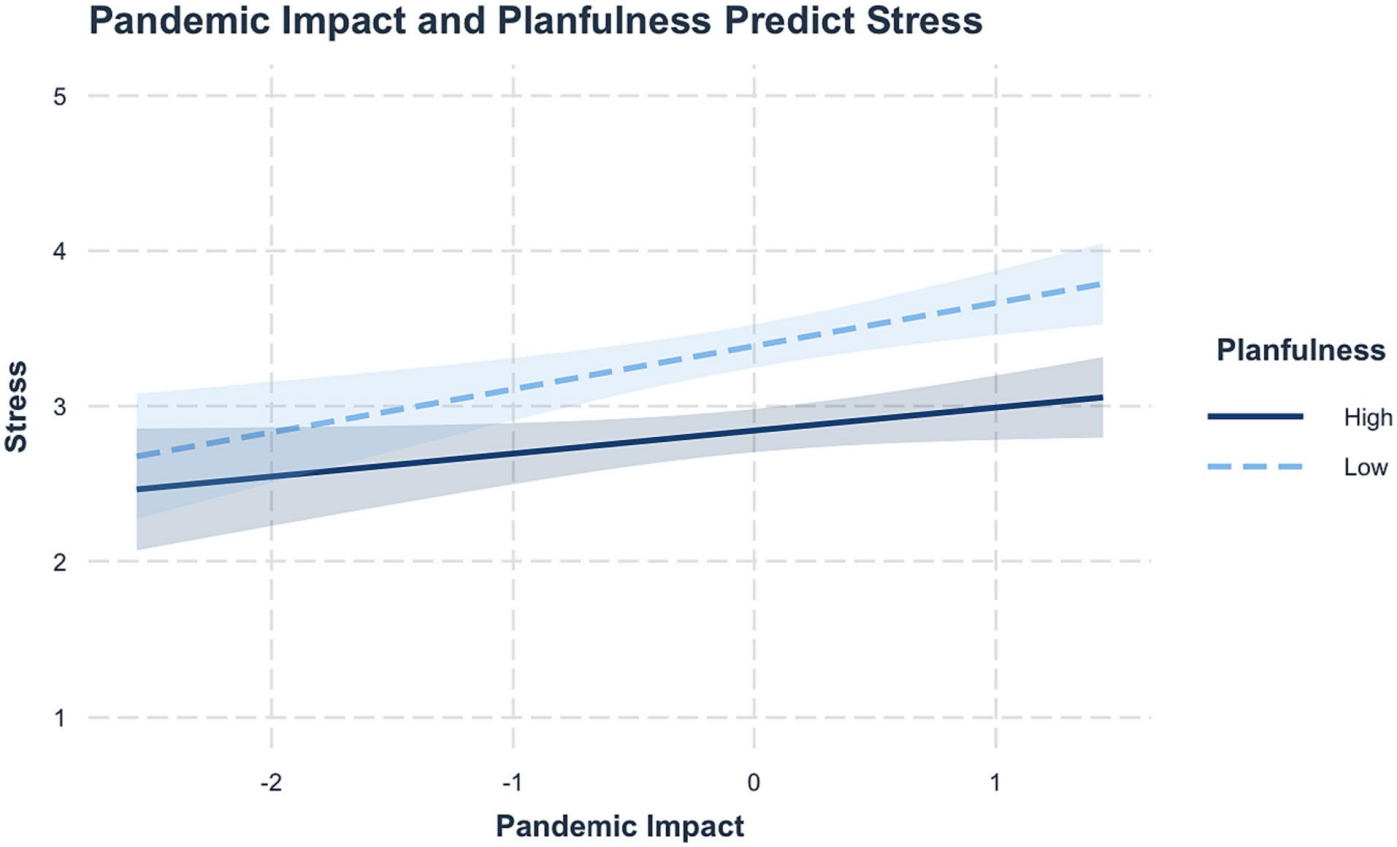
Pandemic impact and planfulness predicting stress. Pandemic impact and planfulness are mean-centered, and shaded regions represent 95% CI. High pandemic impact was associated with higher stress, and highly planful individuals maintained lower stress than students low in planfulness.

For stress, there were main effects of pandemic impact (*β* = 0.21, *SE* = 0.54, *p* < 0.001) and planfulness (*β* = −0.52, *SE* = 0.95 *p* < 0.001), with high pandemic impact predicting higher stress, and high planfulness predicting lower stress. There was no interaction between pandemic impact and planfulness (*β* = −0.12, *BSE* = 0.10 *p* = 0.21, *R*^2^ = 0.21). High levels of pandemic impact predicted higher levels of stress, and highly planful individuals maintained lower levels of stress than less planful individuals regardless of pandemic impact levels (Figure 3).

Our exploratory mediation analyses found significant but modest indirect effects of planfulness on well-being (*b* = 0.07, 95% BCa CI [0.001, 0.16]) and stress (*b* = −0.05, 95% BCa CI [−0.13, −0.0001]) mediated by goal disruption. Full results from these analyses can be found in the Supplementary material for this manuscript (Supplementary Figures 1, 2).

## 4 Discussion

Planfulness refers to stable individual differences in the tendency to be oriented to the future implications of a behavior, to employ cognitive strategies which contextualize that behavior in terms of one’s goals, and to be mentally flexible in anticipating and adjusting to obstacles to those goals (Ludwig et al., 2018). Previous work has linked planfulness to self-reported and objective measures of goal pursuit (Ludwig et al., 2018, 2019), yet as a newly developing construct, we know little about its association with psychological experiences beyond the goal domain. In this study, we extended examinations of planfulness to probe its relationships with the critical psychological outcomes stress and psychological well-being. We found that students who were higher in planfulness exhibited higher levels of psychological well-being and lower levels of stress, indicating that planfulness not only helps people persist in pursuing their goals, but is also aligned with positive mental health. These findings also provide further evidence that variables related to goal pursuit generally can support well-being. That is, goal progress and goal achievement are related to increased well-being (Brunstein, 1993; Sheldon and Kasser, 1998) and meaning in life (Mohideen and Heintzelman, 2023), and individual differences in goal-related cognition and strategy appear to be as well. For example, writing about one’s life goals is related to increased well-being (King, 2001), and many people engage in “planning” as a coping strategy when faced with a setback (Pigaiani et al., 2020; Eisenbeck et al., 2022).

Furthermore, the COVID-19 pandemic created a unique shared setback context in which to examine the interrelationships between planfulness and these outcomes in the face of extreme challenges. We found that college students higher in planfulness experienced less disruption of their goals in the general context of the COVID-19 pandemic. However, when leveraging participants’ perceptions of the impact the pandemic was having on them personally, high levels of planfulness did not protect against goal disruption among those participants in which the self-reported personal impact of the pandemic was highest. Differences in goal disruption across levels of planfulness were constrained to less severe impacts from COVID-19. It is possible that the pandemic is a strong situation that overwhelms this individual difference that normally promotes goal pursuit, dampening its effects (Cooper and Withey, 2009). Indeed, the uncertain nature of the COVID-19 pandemic made planning difficult: It was unknown when it would worsen or ease, when restrictions would be intensified or be lifted, when a relative or friend would contract COVID-19, and so on. These results point to the fact that while planfulness appears to be a beneficial individual difference for goal outcomes in general, it cannot fully protect against a setback as severe or as prolonged as the COVID-19 pandemic, potentially because this context was hostile to planning and so highly planful individuals are not able to usefully leverage planfulness strategies. However, it is important to note that unlike some previous work which has found mixed outcomes of using planning as a coping strategy (Eisenbeck et al., 2022), higher planfulness did not result in higher stress, so current results indicate that there appear to be no direct negative outcomes associated with high planfulness. To further test the possible buffering influence of planfulness, future work ought to examine these relationships in the context of a more prototypical and less severe setback that does not directly impact one’s ability to engage in planning strategies.

For stress and psychological well-being, however, there were no interactions of pandemic impact and planfulness. The differences in psychological well-being and stress by levels of planfulness were retained even when self-reported perceptions of personal pandemic impact were high. More planful students maintained lower stress and higher psychological well-being than their less planful peers across levels of adversity. These findings suggest that even in extremely difficult contexts in which planfulness does not protect against goal disruption, it still garners personal benefits in terms of psychological health. This provides evidence that planfulness is an important construct beyond goal attainment, even when a person is faced with goal disruption in the midst of a setback.

### 4.1 Limitations and future directions

While our findings suggest that planfulness predicts outcomes outside of pure goal achievement, the current study had a few limitations that offer avenues for future research. First, while our use of self-reported perceptions of pandemic impact was intended to encompass the many different effects of the pandemic, this tactic introduces additional subjectivity into our conceptualization of pandemic-related adversity. This self-reported pandemic impact variable did not differ between participants who reported having tested positive for COVID-19 or relate to the number of people that the participant knew who had contracted COVID-19. It is clear that this measure of pandemic impact captured more than personal experiences with the COVID-19 illness itself, but encounters with other related challenges including financial or educational difficulties. While the self-reported impact variable appropriately captures a breadth of challenges related to COVID-19, it may also be affected by other unmeasured personality differences or coping strategies employed by participants that could modify the observed relationships. Additionally, this is a single-item measure, which did not allow for internal measurement validity. Due to data collection happening in the midst of the pandemic we were unable to use already existing scales to measure this and opted to prioritize face validity and ask participants simply about their perceived subjective experience, rather than trying to generate objective measures that would not fully capture this experience. Similarly, our measure of goal disruption was worded to reflect the impact of the pandemic on disruption of students’ goals, and future work should investigate these effects with a more general self-reported measure of goal-disruption or an objective measure of goal progress. Future research should thus examine planfulness processes in adversity by experimentally manipulating setbacks in controlled laboratory experiments and observing effect differences on general goal and psychological health outcomes by levels of planfulness. Relatedly, the results in our mediation analyses, while significant, were modest and should be replicated (perhaps again with a more domain-general measure of goal disruption).

Another limitation of this study is our use of a small convenience sample. As this study was conducted in a specific moment in time (Fall, 2020 and Spring, 2021) when college courses were being conducted remotely and many social distancing measures were in place, we are unable to collect more data to replicate these effects with a larger sample. Although it was quite racially diverse, our small sample also limits our ability to conduct more nuanced analyses. Specifically, there is strong evidence of the differential impacts of the pandemic among different racial and ethnic groups (Centers for Disease Control and Prevention, 2022). While we found no difference in the pandemic impact variable or any of the dependent variables between White and Black participants or Latinx vs. non-Latinx participants, our study was likely underpowered to detect any such differences. This is an area that future research can explore.

As the science of planfulness continues to develop, there are many additional avenues for future research to build on the current work. First, additional research is needed to further investigate potential mechanisms accounting for the links between planfulness and stress and psychological well-being in both standard and adversity-ridden contexts. While the current study demonstrates that these relationships exist, future work can further uncover why they exist. One possibility is that planfulness impacts well-being and stress due to its relationship with goal progress and achievement. As a step toward investigating this idea our exploratory mediation analyses indicated that goal disruption mediates the relationship between planfulness and well-being and stress (Supplementary Figures 1, 2). Researchers could employ experience sampling methodologies to examine the extent to which planfulness-related actions of participants in real-life contexts relate to outcomes in the goal and psychological health domains. Another promising direction for future research is exploring interventions to foster planfulness. While it is a relatively stable individual difference, manipulations designed to promote planfulness could give individuals, and students in particular, additional tools for engaging with long-term goals and maintaining psychological wellness even in the face of setbacks.

### 4.2 Conclusion

The COVID-19 pandemic was an unprecedented disruption, leading to significant declines in mental health, and well-being. This context provided a unique opportunity to investigate factors which may have played a protective role against these adverse effects. The results from the present study demonstrate the importance of planfulness for progress toward desired ends as well as for psychological well-being and stress reduction in the midst of major life challenges. While future research will continue to show how the pandemic is similar to, and different from, other more common stressors, our results show that being high in planfulness confers many benefits for goal progress and mental wellness. Planfulness is associated with lower perceived stress, higher levels of psychological well-being, and lower disruption of goals. Furthermore, even when facing a setback as disruptive as a global pandemic, highly planful students exhibited lower levels of stress and higher levels of psychological well-being, pointing to the important value of planfulness for maintaining positive psychological health amid adversity.

## Data availability statement

The raw data supporting the conclusions of this article will be made available by the authors, without undue reservation.

## Ethics statement

The studies involving humans were approved by Rutgers University Institutional Review Board. The studies were conducted in accordance with the local legislation and institutional requirements. The Ethics Committee/Institutional review board waived the requirement of written informed consent for participation from the participants or the participants’ legal guardians/next of kin because the consent process and data collection for this study took place online. Participants were members of Rutgers-University courses and obtained extra credit for voluntarily participating in the online surveys. All survey items were self-report, no academic records were pulled for any participants.

## Author contributions

WA, ET, and SH contributed to the conception and design of the study. WA performed the statistical analyses and wrote the first draft of the manuscript. All authors contributed to the manuscript revision, read, and approved the submitted version.
