# Supplementary material for: The role of planfulness for well-being, stress, and goal disruption during COVID-19
Source: Front Psychol. 2024 Feb 8;15:1224451. doi: 10.3389/fpsyg.2024.1224451 (PMC10881737; doi:10.3389/fpsyg.2024.1224451)
Supplement: Supplementary file 1 [file Table_1.DOCX]

**Goal Disruption as a Mediator**

Exploratory analyses investigated whether the relationships between planfulness and stress and well-being might be explained through the relationship between planfulness and goal disruption. Put simply, does higher planfulness relate to less disruption of one’s goals, which in turn leads to higher psychological well-being and lower stress? To test this we used Hayes’ PROCESS macro for R (Hayes, 2022) which tests for the presence of mediation using bootstrapped 95% confidence intervals.

For well-being, there was a significant but modest indirect effect of planfulness on well-being that was mediated by goal disruption, *b* = .07, 95% BCa CI [.001, .16]. This is indicated by the confidence interval around the indirect effect not containing 0. Higher planfulness is related to less disruption of one’s goals which in turn is related to higher psychological well-being (Supplementary Figure 1.).

**
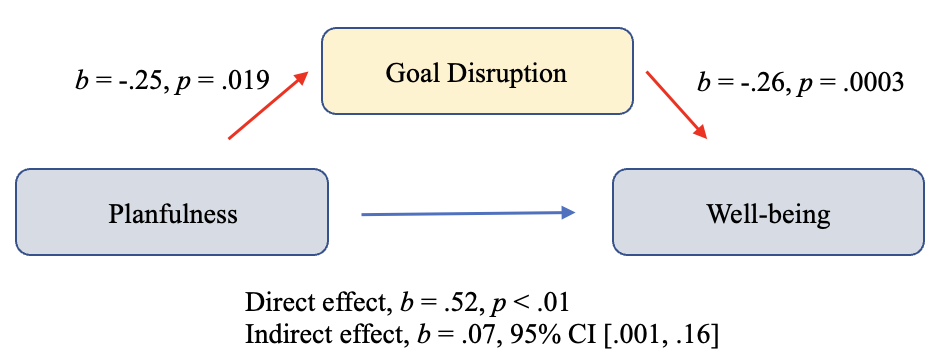
**.

**Supplementary Figure 1.** **Planfulness as a predictor of well-being, mediated by goal disruption.** The confidence interval for the indirect effect is a bootstrapped confidence interval based on 5000 samples. Higher planfulness is related to less disruption of one’s goals which in turn is related to higher psychological well-being. Red arrows indicate negative effects and blue arrows indicate positive effects.

For stress, there was a significant but modest indirect effect of planfulness on stress mediated by goal disruption, *b* = -.05 , 95% BCa CI [-.13, -.0001]. This is indicated by the confidence interval around the indirect effect not containing 0. Higher planfulness is related to less goal disruption, which in turn is related to lower stress (Supplementary Figure 2).
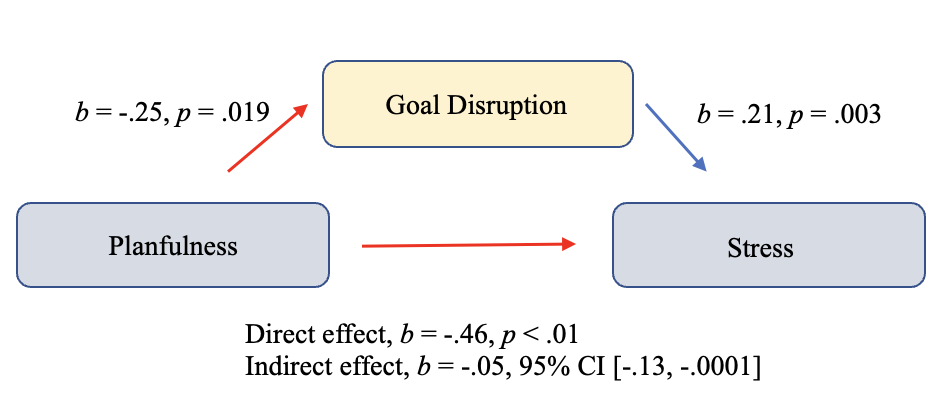


**Supplementary Figure 2.** **Planfulness as a predictor of stress, mediated by goal disruption.** The confidence interval for the indirect effect is a bootstrapped confidence interval based on 5000 samples. Higher planfulness is related to less disruption of one’s goals which in turn is related to lower stress. Red arrows indicate negative effects and blue arrows indicate positive effects.

**References**

Hayes, A. F. (2022). *Introduction to mediation, moderation, and conditional process analysis: A regression-based approach* (Third edition). The Guilford Press.
